# A retrospective quality assessment of pre-hospital emergency medical documentation in motor vehicle accidents in south-eastern Norway

**DOI:** 10.1186/1757-7241-19-20

**Published:** 2011-03-31

**Authors:** Trine Staff, Signe Søvik

**Affiliations:** 1 Department of Research, Norwegian Air Ambulance Foundation, Drøbak, Norway; 2 Norwegian Centre for Pre-hospital Emergency Medicine (NAKOS), Oslo University Hospital, Norway; 3 Department of Anaesthesiology, Akershus University Hospital, Oslo, Norway; 4 University of Oslo, Faculty Division Oslo University Hospital, Kirkeveien, Oslo, Norway

## Abstract

**Background:**

Few studies have evaluated pre-hospital documentation quality. We retrospectively assessed emergency medical service (EMS) documentation of key logistic, physiologic, and mechanistic variables in motor vehicle accidents (MVAs).

**Methods:**

Records from police, Emergency Medical Communication Centers (EMCC), ground and air ambulances were retrospectively collected for 189 MVAs involving 392 patients. Documentation of Glasgow Coma Scale (GCS), respiratory rate (RR), and systolic blood pressure (SBP) was classified as exact values, RTS categories, clinical descriptions enabling post-hoc inference of RTS categories, or missing. The distribution of values of exact versus inferred RTS categories were compared (Chi-square test for trend).

**Results:**

25% of ground and 11% of air ambulance records were unretrieveable. Patient name, birth date, and transport destination was documented in >96% of ambulance records and 81% of EMCC reports. Only 54% of patient encounter times were transmitted to the EMCC, but 77% were documented in ground and 96% in air ambulance records. Ground ambulance records documented exact values of GCS in 48% and SBP in 53% of cases, exact RR in 10%, and RR RTS categories in 54%. Clinical descriptions made post-hoc inference of RTS categories possible in another 49% of cases for GCS, 26% for RR, and 20% for SBP. Air ambulance records documented exact values of GCS in 89% and SBP in 84% of cases, exact RR in 7% and RR RTS categories in 80%. Overall, for lower RTS categories of GCS, RR and SBP the proportion of actual documented values to inferred values increased (All: p < 0.001). Also, documentation of repeated assessment was more frequent for low RTS categories of GCS, RR, and SBP (All: p < 0.001). Mechanism of injury was documented in 80% of cases by ground and 92% of cases by air ambulance.

**Conclusion:**

EMS documentation of logistic and mechanistic variables was adequate. Patient physiology was frequently documented only as descriptive text. Our finding indicates a need for improved procedures, training, and tools for EMS documentation. Documentation is in itself a quality criterion for appropriate care and is crucial to trauma research.

## 1. Background

In trauma research, there are few studies of documentation quality in the pre-hospital emergency medical services (EMS) that deliver care during "the golden hour". Important information on the mechanism of injury and initial patient physiology can only be gathered at the trauma scene, where several emergency services with differing objectives interact. Trauma from motor vehicle accidents (MVAs) is common, and these accidents place a great burden on society, both personal and economical. In Norway, which has a population of 4.9 million, the number of registered deaths from MVA in the study year (2005) was 224 [1].

The World Health Organization has stated that there is a need for studies on decisive factors in trauma outcomes, for prevention, education, and health planning purposes [2,3]. In Scandinavia, great efforts have been made in recent years to improve early trauma care. Guidelines have been developed for pre-hospital airway management [4], for massive bleeding in trauma patients [5], and for uniform reporting of data on major trauma [6]. Still, an ongoing debate over the required skills levels, procedures, methodology, and variables to be reported by EMS delay the implementation of uniform agreements [7-10].

This study was part of a cross-sectional MVA study evaluating whether patient injury pattern and severity is associated with e.g. accident type, mechanical distortion of the vehicle, unrestrained objects in the vehicle, and seat-belt use. Here, we hypothesized that the variation in documentation tools, personnel training and patient selection between EMS services would affect the quality of pre-hospital documentation. Our retrospective study sought to assess the completeness and quality of EMS documentation of key logistic, physiologic, and mechanistic variables in MVAs from a trauma research perspective. To evaluate the documentation of patient consciousness, respiration and circulation we chose to assess the documentation rate of Glasgow Coma Scale (GCS), respiratory rate (RR) and systolic blood pressure (SBP), which are used to calculate the Revised Trauma Scale (RTS). When neither exact values nor RTS categories were documented, we evaluated whether some clinical descriptions or check box categorizations in EMS reports could be used to post-hoc infer RTS categories for GCS, RR and SBP. Inference of categorical values introduces uncertainties in research data but greatly reduces data loss due to missing values.

EMS documentation is often performed in chaotic and complex settings: in the dark, rain, and cold, under time pressure, and sometimes under threat to personal safety. Still, all research on pre-hospital trauma care, the use of EMS, and mechanism of injury (MOI) in MVAs depends heavily on this documentation. A potential consequence of our study could be to increase the EMS services' attention to documentation quality and to highlight the benefit of a uniform, exact EMS reporting standard from the perspective of using such data for trauma research.

## 2. Methods

This was a retrospective, observational, cross-sectional study of the completeness and quality of EMS documentation in MVAs. Completeness was studied by assessing documentation rate. Quality of physiologic data was studied by assessing whether variables were reported as exact figures, as RTS categories, or through broadly defined categories or free text precise enough to allow post-hoc inference of RTS categories.

### 2.1. Setting

Data were collected from Dec. 1, 2004 to Jan. 31, 2006 from MVAs in nine counties in south-eastern Norway, covering 136,000 square kilometres with a population of 2.7 million people. Seven Emergency Medical Communication Centres (EMCCs), 13 police districts, 99 ground ambulance stations, five air ambulance bases, and one Air Force search and rescue helicopter were active in the study area. The ground and air ambulance systems were both part of the specialised health service. The ground ambulances were staffed with emergency medical technicians (EMTs) and/or paramedics. The air ambulances were staffed with a pilot, an anaesthesiologist, and a rescue professional.

### 2.2. Data collection

Study approval and appropriate permits were obtained from the Regional Committees for Medical and Health Research Ethics, the Norwegian Directorate of Health and Social Affairs, the Norwegian Data Inspectorate, and the Attorney General. For all cases, we attempted to retrospectively collect and review police reports, EMCC reports, and ground and air ambulance records completed by EMTs, paramedics, or anaesthesiologists. Arrival records from hospitals or Local Emergency Medical Centre (LEMC) were collected in cases where EMS records could not be retrieved, because hospital arrival records often cite information from the oral report routinely given by EMS personnel when handing over a patient (Table 1).

**Table 1:** Data collection instrument

Accident number	**Motor vehicle number**:	Patient number:	
**Dispatch criterion**	Member from research accident team alerted and dispatched by the EMCC	Y N	
Motor vehicle accident			
- suspicion of serious injury or death			

**Patient record retrieved**	Police	Y N	
	EMCC	Y N	
	Ground ambulance	Y N	
	Air ambulance	Y N	
In case of missing EMS records	Hospital/LEMC	Y N	

**Personal identification data**	Patient First name	Y N	
	Patient Family name	Y N	
	Birth date (6-digit)	Y N	
	Social security number (11-digit)	Y N	

**Logistic variables**	EMCC Unique Identifier Number	Y N	
	Accident date	Y N	
	Transport destination	Y N Wrong	
	**Patient encounter times**		
	Alarm at EMCC	Y N	
	Ground/Air ambulance departure from station		Y N
	Ground/Air ambulance arrival on scene		Y N
	Ground/Air ambulance departure from scene		Y N
	Ground/Air ambulance arrival at destination		Y N

**Glasgow coma scale (GCS)**	GCS exact value documented	Y N	
	GCS RTS category	4 3 2 1 0	
	GCS RTS category inferred	Y N	
	GCS assessments repeated every 20 min	Y N	

**Respiratory rate (RR)**	RR exact value documented	Y N	
	RR RTS category	4 3 2 1 0	
	RR RTS category inferred	Y N	
	RR assessments repeated every 20 min	Y N	

**Systolic blood pressure (SBP)**	SBP exact value documented	Y N	
	SBP RTS category	4 3 2 1 0	
	SBP RTS category inferred	Y N	
	SBP assessments repeated every 20 min	Y N	

**Variables relevant for mechanism of injury (MOI)**	≥ 2 MOI factors documented		Y N
	MOI reported as	Check boxes	Free text
	Patient location in vehicle	Driver	
		Front passenger	
		Rear passenger	

Y N indicates whether variables were documented or not (Y = yes, N = no), for each relevant service

Data were requested from those responsible for administering the archives in the various services. Letters of request to the different institutions were sent up to three times in cases of no response. When ambulance records could not be retrieved from the EMS, we searched the in-hospital electronic patient record for scanned-in copies. When a large number of ambulance records were missing from any one EMS service, an additional search in the hospital paper archives was performed.

### 2.3. Eligibility criteria

Based on the dispatch criterion "motor vehicle accident - suspicion of serious injury or death," the EMCC notified one of the six research assistants engaged in our project (experienced paramedics). The research assistants were equipped with a uniformed motor vehicle that had permission to function like an emergency vehicle with light-and-siren response for the study purpose only.

This study of documentation quality was part of a cross-sectional MVA study evaluating whether patient injury pattern and severity was associated with e.g. accident type, mechanical distortion of the vehicle, unrestrained objects in the vehicle, and seatbelt use. An MVA was included in the study only if one or more patients were transported by the EMS to a hospital or a LEMC and the research assistant was able to collect mechanical data from the motor vehicle. Collection of mechanical data was performed in collaboration with the police and the Norwegian Public Road Accident groups and was both elaborate and labour demanding. Therefore, the number of MVAs included in this study did not reflect the true number of MVAs occurring in the study area.

### 2.2. Data sources and measurements

For each accident there was one police report. In accidents occurring on the border between different EMCC regions, up to three EMCC records could exist per accident. These were handled as one record for each patient during data analysis. Each MVA could involve several patients, and since each patient could be cared for by more than one ambulance (ground and air), the sum of ambulance records could exceed the total number of patients.

Identification of involved patients was primarily performed through police and EMCC reports. Associated ground and air ambulance records were identified on the basis of the EMCC record's Unique Identifier, accident date, ambulance vehicle number, and the ID of pre-hospital personnel. Hospital arrival records were collected on the basis of patient ID. Incomplete police, EMCC, and EMS documentation could therefore lead to non-inclusion of patients. The total number of patients thus reflects all patients ultimately identified by name and social security number, which includes birth date.

Ambulance records were mainly filled in prospectively and completed by the time the patient was handed over to the receiving hospital or LEMC. In contrast, police reports were completed retrospectively over a period of days, on the basis of investigations and witness interviews.

While there is no standard Norwegian ambulance record, six of the nine counties used the same EMS standard operating procedures, the Medical Operative Manual (MOM). The study variables selected (Table 1) were based on core data listed in the Norwegian national health legislation, the MOM, the Norwegian Index of Emergency Medical Assistance used by all EMCCs, and the Utstein Guidelines for Major Trauma [6,11-14]. These state that ambulance records should document the date of the accident, full patient identification, patient encounter times, physiologic measurements, and relevant background information for each patient, such as the mechanism of injury in the MVA.

**Identification data **gathered included patient first name, family name, birth date, social security number (which includes birth date), and the EMCC-generated Unique Identifier number for each accident. Police, EMCC and ground ambulance report eleven- digit social security number, while air ambulance report birth date only. All EMS services transporting patients from the same accident mark their records with this EMCC Unique Identifier.

**Pre-hospital patient encounter times **are not documented by the police, but the EMCC automatically records the time points when the alarm call is received and when an ambulance is dispatched. These time points normally are electronically transmitted to the ground and air ambulance services, which typically directly transmit back into the EMCC record the times of (1) departure from the station, (2) arrival on-scene, (3) departure from the scene, and (4) arrival at the hospital or LEMC. In addition, there are fields for manually recording the same time data in the ambulance records. We registered the frequencies of completion of these patient encounter times, both in the electronic EMCC records and in the ambulance records. Patient care time was defined as the time interval from EMS arrival on-scene to arrival at the hospital/LEMC. Documentation of transport destination was registered as present, missing, or wrong (Table 1).

**Core physiologic data **include documentation of patient consciousness, respiration, and circulation. The MOM for the ground ambulances in the study area does not specify a required level of detail or time resolution for the documentation of physiologic variables.

Glasgow Coma Scale (GCS) score, respiratory rate (RR), and systolic blood pressure (SBP) are considered key physiologic variables and are used to calculate the Revised Trauma Score (RTS) [6,14-16]. As our criterion for whether the physiologic EMS documentation would be useful for trauma research we therefore registered whether GCS, RR and SBP was documented in the EMS records as (1) exact values or as (2) RTS categories (0-4) (See Table 2) [15,16]. If no such GCS, RR, or SBP documentation existed, we evaluated whether clinical descriptions of patient consciousness, respiration, and circulation in check boxes or free text fields were sufficient to reasonably post-hoc infer an RTS category. Table 2 illustrates how clinical descriptions in ground and air ambulance records were used to post-hoc infer an RTS category value. When patient descriptions were too ambiguous to reasonably infer a RTS category, data were categorised as missing. The classification was performed by one of the authors (TS) on the basis of published clinical categories [6,14-16].

**Table 2:** Clinical descriptions used to infer RTS categories for GCS, RR, and SBP

	Glasgow Coma Scale		Respiratory rate		Systolic Blood Pressure	
	
RTS Category	Exact values	Clinical descriptions used to infer RTS	Exact values	Clinical descriptions used to infer RTS	Exact values	Clinical descriptions used to infer RTS
**4**	**13-15**	Awake Oriented Fully conscious	**10-29**	Normal, unaffected	**>89**	Good radial pulse
**3**	**9-12**	Confused, Somnolent Disoriented, Abnormal reflex movement	**>29**	Fast hyperventilation	**76-89**	**-**
**2**	**6-8**	**-**	**6-9**	Slow, insufficient	**50-75**	**-**
**1**	**4-5**	**-**	**1-5**	**-**	**1-49**	**-**
**0**	**3**	Deeply unconscious Unawake, no motor response, no speech	**0**	No respiration	**0**	No palpable pulse No carotic pulse No circulation Flat ECG curve

Empty cells: No clinical descriptions were considered adequate to reasonably infer these values of RTS categories

We also registered whether GCS, RR, and SBP assessments, or clinical descriptions of consciousness, respiration, and circulation, were repeated at least every 20^th ^minute during patient care time. When patient care time lasted less than 20 minutes, one documented assessment of consciousness, respiration, and circulation data was considered sufficient to be logged as "Repeated". For records with missing patient encounter times or missing GCS, RR or SBP data, the data fields for repeated physiologic assessments were coded as missing.

**Mechanism of injury: **For legal purposes, the police attempts to identify the driver of each vehicle involved in an MVA. The location in the car of the other injured persons is only recorded as front or rear seat occupants. In contrast, EMS services attempt to record the mechanism of injury for all patients. According to local procedures and international Utstein Guidelines, key variables important in determining mechanism of injury (MOI) in MVA patients include high vehicle speed, patient location in the vehicle, cabin intrusion, ejection from vehicle, death in the same compartment, entrapment, impact direction, and vehicle roll-over. We registered whether ambulance records documented two or more of the factors describing MOI of the accident.

### 2.3. Data analysis

Data from ground and air ambulance records were compared with the information available from police and EMCC reports. Descriptive statistics and chi-square tests for two-way analyses were performed in SPSS for Windows v.18. Box plots illustrate the 25^th^-75^th ^percentile (box), bars represent the 90^th ^percentile.

We hypothesised that documentation quality might be better on ambulance missions with more severely injured patients. Also, both mission profiles and personnel training was heavily skewed in our material. The paramedic-staffed ground ambulances transport a broad selection of patients, while the anaesthesiologist-staffed air ambulance is dispatched when information in the alarm call or from personnel already on-scene indicates that patients are likely to be severely injured. We therefore used a chi-square test for trend [17] to compare the distribution of RTS categories for GCS, RR and SBP (five-level ordinal categorical variables) between patients with documented exact values or RTS categories and patients where RTS categories were inferred post-hoc. By the same method, we evaluated whether poorer RTS category was associated with improved time resolution of physiologic measures (higher frequency of repeated assessments).

## 3. Results

### 3.1. Demographic data

We included 190 accidents involving 338 motor vehicles and 618 persons. Of these, 226 persons were excluded because they were dead on-scene (n = 62), not injured (n = 160), or transported by means other than EMS (n = 4). Documented patient destination was a hospital in 362 cases and an LEMC in 30 cases.

For the 392 patients included in the study, the number of successfully retrieved records is listed in Table 3. EMS records could not be retrieved for 25% of patients transported by ground and 11% of patients transported by air ambulance. For these 86 patients, we recovered 76 hospital arrival records.

**Table 3:** Retrieved pre-hospital records by care provider

	Police	EMCC	Ground ambulance	Air ambulance
**Identified patients**	**392**	**392**	**308**	**84**

**Retrieved records**n (%)	368 (94)	392 (100)	231 (75)	75 (89)

EMCC: Emergency Medical Communication Centre

All police reports were constructed using the same template. All EMCC and air ambulance services also used national, standardised records. In contrast, seven different ground ambulance record templates were in use in the nine counties. Three counties used the same template, while in one county, two different templates were used. Six of seven record templates were single-paged, whereas one was two-paged.

### 3.2. Patient ID

Patient identification data varied between services. Patients were identified by first and family name in 97% of police, ground and air ambulance records, and in 81% of EMCC records. Eleven-digit social security number (including birth date) was documented in 380 of 392 (97%) police records, 300 of 401 (74%) EMCC records, 138 of 231 (60%) ground ambulance records, and 17 of 75 (23%) air ambulance records. Birth date only was documented in 20 of 401 (5%) EMCC records, 83 of 231 (36%) ground ambulance records, and 54 of 75 (73%) air ambulance records. All in-hospital documentation records contained patients' first and family name and social security number.

### 3.3. EMS logistics

Date of accident was documented in all police, EMCC, ground and air ambulance records. Most ground (87%) and air ambulance (99%) records included correct EMCC Unique Identifiers. Transport destination was incorrectly documented in six EMCC records (1.5%) and was missing in 18% of cases. There were no incorrect destinations in any ground or air ambulance records, but the destination was missing in 6% of cases.

Documentation of patient encounter times varied between services (Table 4). Time points originating at the EMCC (time of alarm to EMCC and of ambulance departure) were documented for all missions. In contrast, about half of the time points that should have been electronically transmitted from the EMS to the EMCC record were missing. Ground ambulances documented three of four patient encounter times in their own paper records, whereas this documentation was almost complete in air ambulance records.

**Table 4:** Patient encounter times documented

	EMCC		Ground ambulance		Air ambulance	
	N = 392		N = 231		N = 75	
	n (%)		n (%)		n (%)	
**Alarm at EMCC**	392	(100)	173	(75)	53	(71)
**EMS departure**	392	(100)	177	(77)	72	(96)
**EMS arrival on-scene**	219	(56)	172	(75)	72	(96)
**EMS departure from scene**	205	(52)	163	(71)	72	(96)
**EMS arrival at destination**	211	(54)	193	(84)	73	(97)

Results are listed as number (%) documented.

EMCC: Emergency Medical Communication Centre. EMS: Ground or air ambulance

### 3.4. Physiologic measurements

All ambulance records included fields for reporting consciousness, respiration, and circulation. The various templates included charts where repeated physiologic measurements could be documented at specified time points, check boxes for RTS categories, check boxes for broadly defined categories of physiologic variables, and free text fields. All record templates offered more than one alternative for documenting patient physiology and all prompted repeated measurements by providing repeating fields. Table 5 shows the frequency of various documentation alternatives in the seven ground ambulance templates. The air ambulance template contained fields for free text, open fields for entering the RTS categories of RR, SBP, and eye, verbal, and motor components of the GCS scale, and a medical chart for repeated measurements of heart rate and blood pressure.

**Table 5:** Alternatives for documenting patient physiology in seven different ground ambulance record templates

	Exact values		RTS categories		Broadly defined categories	Clinical descriptions
	
Patient physiology	Chart		Check boxes		Check boxes	Free text field
**Consciousness**	GCS	6/7	GCS	0/7	3/7	7/7
**Repeated field**		3		0	1	

**Respiration**	RR	4/7	RR	3/7	5/7	7/7
**Repeated field**		4		1	2	

**Circulation**	SBP	7/7	SBP	3/7	5/7	7/7
**Repeated field**		7		1	2	

Results are frequencies of the various field types for documentation of physiology among seven ground ambulance record templates. GCS: Glasgow Coma Scale, RR: Respiratory rate, SBP: Systolic blood pressure

Table 6 shows documentation of GCS score, RR, and SBP in ground and air ambulance records. Exact values or RTS categories of GCS, RR, and SBP were documented in 48-64% of cases for ground ambulances and 87-89% of cases for air ambulances. GCS and SBP were almost always documented as exact values. In contrast, RR was rarely reported as breaths/minute, instead RTS category for RR was documented. Post-hoc inference of RTS categories from clinical descriptions in free text and various check boxes was possible for GCS score and RR in almost all of the rest of the ground ambulance records and in one of five records for SBP.

**Table 6:** Patient physiology documented

	Ground ambulance		Air ambulance		Hospital record only*	
	N = 231		N = 75		N = 76	
Patient physiology	n (%)		n (%)		n (%)	
**Glasgow coma scale**						
**Exact values**	110	(48)	67	(89)	8	(11)
**RTS category documented**	0		0		0	
**RTS category inferred**	113	(49)	8	(11)	64	(84)
Repeated**	158	(68)	71	(95)	(11)	(15)

**Respiratory rate**						
**Exact values**	23	(10)	5	(7)	0	
**RTS category documented**	124	(54)	60	(80)	0	
**RTS category inferred**	59	(26)	4	(5)	38	(50)
**Repeated**	109	(47)	60	(80)	1	

**Systolic blood pressure**						
**Exact values**	122	(53)	63	(84)	5	(7)
**RTS category documented**	10	(4)	4	(5)	0	
**RTS category inferred**	47	(20)	4	(5)	32	(42)
**Repeated**	103	(45)	68	(91)	3	(4)

Repeated: Documentation of repeated assessment of the variable at least every 20^th ^minute. EMCC: Emergency Medical Communication Centre. *Hospital arrival records were evaluated when ground and air ambulance records were missing. **Includes free text descriptions of change (or no change) in conscious level.

**Figure 1:**
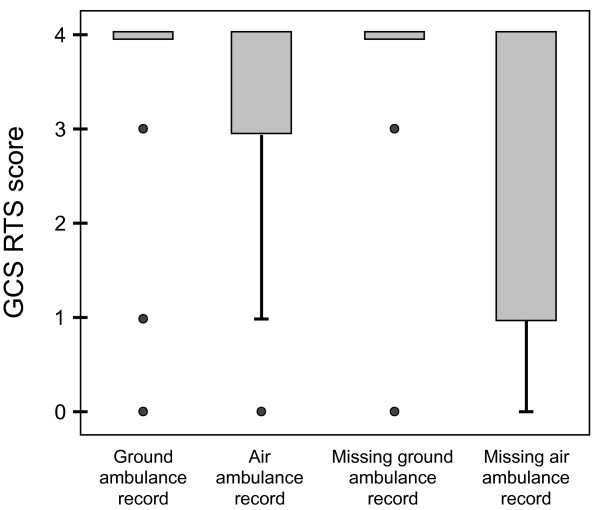
**Distribution of GCS RTS score in patients transported by ground and air ambulance**. Box plot of GCS RTS scores in patients from motor vehicle accidents documented in ground ambulance records (n = 231), air ambulance records (n = 75), and in-hospital arrival records where ground (n = 61) and air (n = 15) ambulance records were missing. Patients transported by air ambulance had poorer GCS RTS scores than those transported by ground. Notably, patients transported by air with missing ambulance records displayed the poorest GCS RTS scores.

GCS RTS categories were lower in patients transported by air ambulance compared to those transported by ground ambulance (Figure 1). Tracheal intubation on-scene was performed in 35% of patients transported by air and in 1% of patients transported by ground ambulance (p < 0.001).

With decreasing patient RTS category (indicating more severe injury), documentation of exact values or RTS category became more frequent and the need to infer values declined (Chi square test for trend, GCS score (p < 0.001), RR (p < 0.001), and SBP (p < 0.001)).

Repeated documentation was also more common with decreasing patient RTS category of GCS score (p < 0.001), RR (p < 0.001) and SBP (p < 0.001). In ground ambulance records a large fraction of the patients were described in free text as "fully awake and oriented" (i.e. with an inferred GCS category of 4) on arrival on-scene and "stable, unaltered throughout transport". As a consequence, GCS score category was the variable most frequently documented repeatedly in the ground ambulance (Table 6). In the air ambulance records exact values of GCS score and SBP, and RTS categories for RR were repeated to an even higher degree (Table 6).

Records with repeated GCS documentation and records without repeated GCS documentation had similar patient care times [median 38 min (range 6-113 min) vs. 44 min (range 1-88 min), Mann-Whitney-U test p = 0.13]. Not repeating GCS documentation was associated with not documenting patient care times (chi-square test p < 0.0001).

### 3.5. Mechanism of Injury

Four out of seven ambulance record templates included check boxes for reporting MOI. The remaining three templates had free text fields but did not prompt reporting MOI. Three templates had check boxes for frontal intrusion, patient ejection from vehicle, death in the same compartment, and patient entrapment. One ambulance template had additional check boxes for high vehicle speed, patient location in vehicle, impact direction, and roll-over. Air ambulance records and hospital records documented MOI through written text only.

Two or more MOI factors were reported in 184 out of 231 (80%) ground ambulance records, in 69 out of 75 (92%) air ambulance records, and in 53 out of 76 (70%) hospital arrival records. The location of the patient in the motor vehicle was documented to a similar degree by the different pre-hospital care providers (Table 7).

**Table 7:** Patient location in motor vehicle documented

	Ground ambulance		Air ambulance		Hospital record only	
	N = 231		N = 75		N = 76	
Patient location	n (%)		n (%)		n (%)	
**Driver**	109	(47)	41	(55)	26	(34)
**Passenger front seat**	27	(12)	12	(16)	14	(18)
**Passenger back seat**	7	(3)	1		6	(8)

**Sum of patients located**	143	(62)	54	(72)	46	(60)

Results are listed as number (%) documented. EMCC: Emergency Medical Communication Centre. *Hospital arrival records were evaluated when ground and air ambulance records were missing.

## 4. Discussion

This retrospective study sought to assess the completeness and quality of EMS documentation of key logistic, physiologic, and mechanistic variables in MVAs from a trauma research perspective. We hypothesized that the variation in documentation tools, personnel training and patient selection between EMS services would affect the quality of pre-hospital documentation. The present investigation highlights the lack of a uniform ambulance record template in the Norwegian EMS. The varying level of detail and time-resolution in the documentation implies a need for more uniform protocols.

A strength of our study is its retrospective design, which precludes the Hawthorne effect and thus provides realistic findings regarding EMS documentation practice. To evaluate the EMS documentation of physiologic values in terms of its usefulness for trauma research, we assessed the documentation rate of exact values and of RTS categories (0 - 4) of GCS score, RR, and SBP. We found that a considerable fraction of ambulance records did not report either exact values or RTS categories. Similarly, in-hospital records from trauma centre emergency departments also frequently have missing RTS data [18]. We found that post-hoc inference of RTS categories was quite often possible when exact values were missing, using clinical descriptions of patient consciousness, respiration, and circulation. By this method, almost all ground and air ambulance records would yield some physiologic data useful for research.

**The method of inferring RTS categories **from descriptive categories or free text introduces a number of uncertainties. First, the variables described in the records were often proxy variables for the RTS variables, e.g. "good radial pulse" was used to approximate blood pressure. Secondly, we decided that only some levels of the RTS categories could be reasonably inferred from clinical descriptions in EMS reports. This resulted in "inferred RTS scales" of poorer resolution than the actual five-level RTS category scales (Table 2). Third, a degree of subjective interpretation is obviously involved when inferring categories from free text descriptions. This subjectivity leads to increased intra- and inter-rater variability. Still, the method of inferring RTS values greatly reduces data loss due to missing values, and it is the recommended method by the expert panel that formed the basis for The Utstein Template for uniform reporting of data following major trauma [6]. Raw values presented as continuous data are of course preferable when obtainable [6]. High-quality core data are imperative for quality assessment and improvement in EMS services and are essential for research on pre-hospital trauma care.

**The choice of GCS score, RR, and SBP as physiologic variables of interest **is not self-evident. The Pre-Hospital Trauma Life Support (PHTLS) concept underscores clinical observations such as level of consciousness, peripheral pulse pressures, and capillary refill as triggers for intravenous volume treatment. In our material, these variables were often documented via check boxes or free text. In contrast, the Advanced Trauma Life Support (ATLS) concept and much of trauma research is heavily based on the RTS variables, and anaesthesiologists are trained to document patient pulse, SBP, and respiratory data frequently.

**Our retrospective study yielded incomplete data sets**. While no EMCC electronic reports were missing, one quarter of ground ambulance paper records and one tenth of air ambulance paper records could not be retrieved. The organisation of the ambulance paper archives in the present study often reflected the nature of the service as a transport provider, i.e. records were archived by mission date or by ambulance car number. Later organisational changes in the services could then result in difficulties in retrieving records for specific patients. Similar challenges with data acquisition and gaps in documentation have been reported from other EMS systems [19-21]. Still, trauma research is often based on aggregated data from single centres or registries, and the percentage of missing values is seldom presented. Missing records introduce bias in medical research, as the amount of missing information seems to be greater in complex cases [20]. Our cross-sectional study of data from EMS service providers, hospitals and LEMCs in nine different counties provides a more complex perspective than studies from a single EMS service or trauma centre. Figure 1 illustrates how patients transported by air ambulance and where pre-hospital records were missing had the poorest GCS RTS categories, possibly representing situations where patient care had been prioritised over written documentation. The large proportion of missing EMS records and data highlights the possibility of bias of unknown size and direction in research on pre-hospital emergency medicine.

In our country, organising the archives by the EMCC Unique Identifier would have been favourable, as this national system would have offered an efficient way to link ground and air ambulance records with EMCC data. In MVAs occurring on the border of different EMCC areas, several EMCC reports are often established. Such reports should be electronically linked to ensure complete accident data. A standardised pre-hospital electronic patient journal would require less space and could provide both efficient and secure storage of such information while allowing for rapid, reliable retrieval of EMS records for clinical audit and research [20].

**Insufficient EMS documentation of trauma patient ID data **has been previously reported in a study from Pakistan[22]. To our knowledge, similar studies have not been published from any Scandinavian or European country. We found that patient name and birth date was documented in >97% of police-, ambulance-, and air ambulance records but in only about 80% of EMCC reports. This discrepancy in documentation of ID data between police, ground and air ambulance services and the EMCC may be due to EMS personnel obtaining full patient ID at the receiving hospital/LEMC but then not communicating these data back to the EMCC.

**Documentation of EMS response times **is important both for clinical audit and research. Evaluation of the time spent from EMCC alarm to EMS arrival on-scene, to departure from scene, and to arrival at the final destination is essential e.g. for decisions on localisation of ground and air ambulance stations and for assessing the pre-hospital patient care. Evaluation of the time from trauma to definitive care is of great importance in MVA, where patients may be in need of rapid transportation to a competent surgical facility. The variable documentation of important time points in patient care across the EMS services is consistent with prior research [23-25]. Some have discussed potential problems resulting from clocks in various parts of the EMS not being synchronised [23,24]. In our study, only one-half of patient encounter times were electronically transmitted back to the EMCC by the EMS services, yet these time points were documented in three out of four ambulance records and in nine out of ten air ambulance records. Similarly, the Office of the Auditor General of Norway reviewed 14 EMCCs and found documentation of patient encounter times to be missing or inconsistent in 16% (range 6 - 47%) of EMCC reports from "light-and-siren" responses [26]. Clearly, studying EMS patient encounter times using EMCC data alone may result in inaccurate conclusions. Data from all available sources should be taken into account. Also, to truly assess efficiency in terms of time usage, the flow of individual patients through the EMS system must be tracked, as one ambulance crew may be the first to arrive on scene, while another crew may eventually transfer the patient to the hospital.

**The precision level of the documentation of physiologic data **was probably affected by documentation tools, personnel training, and patient selection. Design and layout of data collection forms has been shown to prompt users to record data in a specific way, the documentation rates for prompted items being higher than for non-prompted items [20,27]. In the anaesthesiologist-manned air ambulance service exact GCS scores and SBP measurements were documented in 89% and 84% of records respectively. In contrast, these highly trained and experienced crews seldom reported exact values of RR - the air ambulance record templates only contained fields for RR RTS category, which was reported in 80% of cases. Francis et al. found GCS and RR measurements in 80 - 90% of records and SBP measurements in 70% of records from physician-manned ground ambulances [27]. In contrast, the EMT/paramedic-manned ground ambulances in our study only documented exact values of GCS and SBP in 48% and 53% of records, respectively. Exact RR was documented in 10% and RR RTS category in 54%. One cause of this discrepancy may be that mission profiles were skewed with regard to severity of patient injury (Figure 1). The air ambulance generally transports more critically ill patients [28]. We found that overall, decreasing patient RTS category (more severe injury) was associated with more precise documentation of physiologic data and more frequent documentation of repeated assessments.

**Mechanistic descriptions of the motor vehicle accident **were documented in 80 - 92% of ambulance records, implying that there exists awareness among EMS services that mechanism of injury (MOI) may be an independent risk factor for severe injury in MVA. However, only four out of seven ground ambulance record templates prompted for reporting on MOI, and there was a lack of standardisation of which MOI variables to document and to what level of detail. Patient position in the vehicle was documented in 62% and 72% of the ground and air ambulance records, respectively. Naturally, some MOI factors in MVA may not be easily identified by on-scene EMS personnel busy caring for the patient, especially if the patient is removed from the vehicle prior to ambulance arrival.

**Effects of confounders **like geographic location and personnel training on EMS documentation practice were not explored in this study. However, a cross-sectional survey within the physician-manned pre-hospital services in Scandinavia performed by Kruger et al [21] found a great variation of documentations practices. Secondly, densely populated areas in Norway have been found to have better educated and more experienced ambulance personnel providing patient care [26]. Our study area included both very densely and more sparsely populated areas, and factors such as individual skills, competence, and experience probably also contributed to the variability. To explore this interesting field, a prospective study of EMS documentation practice in relation to the education level and experience of individual EMS personnel would have to be performed.

**Seven different ground ambulance record templates **were in use in the study area, each template with a variety of options for documenting patient physiology (Table 5). Given the variability in record template design and the fact that there was no formal EMS procedure guiding which physiologic variables to document, to what level of detail, and with what time-resolution, the observed differences in practice are unsurprising. Structured, disease-specific assessment fields, such as check-lists, have been shown to increase the quality of both documentation and patient care [29]. However, Francis et al. [27] found that just implementing standard operative procedures with no additional educational or other motivational efforts increased documentation rate in only 5- and 10% of the cases for GCS score and RR, respectively, and not at all for SBP [27]. A central question seems to be whether RTS categories should be prompted for or not in EMS reports. In our material, there was an unexplained difference in practice concerning use of exact values and RTS categories for SBP and RR. An interesting study in the Norwegian EMS ground ambulance system would be to introduce different EMS templates followed by educational- and motivational efforts, and to evaluate the documentation quality after one year. A possible conclusion could be that the data capture would increase by including RTS categories in a template, but probably at the expense of documented exact values. An unfavourable consequence of ambulance records not reporting exact values of GCS, RR and SBP is that it precludes using these data to re-assess the RTS coefficients in a Norwegian trauma population. Continuous reviewing within EMS services is crucial for improving medical documentation.

Our findings imply a need for increased standardisation, clearer procedures, improved training, and evidence-based tools for EMS documentation. Documentation is in itself a quality criterion for appropriate care and is crucial for clinical audit and trauma research.

## 5. Perspectives

The introduction of a national, standardized patient record template for the ambulance service in Norway would lead to more uniform documentation. Prospective studies of how record template design affects documentation rate and quality should be performed. A uniform ambulance record template with explicit field definitions and agreed-upon guidelines for their use would reduce the variability in documentation caused by differing geographical area and personnel competence. The resulting improvement in documentation quality would benefit clinical audit as well as the prospects for trauma research.

During this study, some important aspects of how documentation quality could be improved in the Norwegian EMS service have come to our attention. Targets of action and problems that may need to be resolved are listed below.

• A national, standardized medical operative ambulance manual is needed

• A national, standardized EMS ambulance record template is needed

• All pre-hospital archives should be organized by an EMCC unique identifier

• EMCCs in neighbouring districts need to cooperate and coordinate their documentation. Only one EMCC report should be generated for each accident. Alternatively, all EMCC reports for the same MVA must be electronically linked

• Electronic ambulance records would require less archive space, would provide efficient and secure patient information storage, and allow for rapid, reliable retrieval of data for clinical audit and research

• Patient ID, date of accident and all patient encounter times should be automatically transferred to the EMCC record upon data entry in an electronic ambulance record

## 6. List of abbreviations

ATLS: Advanced Trauma Life Support; EMCC: Emergency Medical Communication Centre; EMS: Emergency medical service; EMT: Emergency medical technician; GCS: Glasgow Coma Scale; LEMC: Local Emergency Medical Centre; MOI: Mechanism of injury; MOM: Medical Operative Manual; MV: Motor vehicle; MVA: Motor vehicle accident; PHTLS: Pre-Hospital Trauma Life Support; RR: Respiratory rate; RTS: Revised Trauma Score; SBP: Systolic blood pressure.

## 7. Competing interests

TS is a research fellow in the Norwegian Air Ambulance Foundation. SS has no competing financial interests to report.

## 8. Authors' contributions

TS was involved in the study conception and design, acquisition of data and data analysis, and the drafting, revising, and final approval of the manuscript. SS was involved in the study conception and design, data analysis, and in the revising and final approval of the manuscript.
